# Supplementary material for: Entropic topography associated with field-induced quantum criticality in a magnetic insulator DyVO4
Source: Sci Rep. 2022 Jan 7;12:56. doi: 10.1038/s41598-021-04389-z (PMC8741807; doi:10.1038/s41598-021-04389-z)
Supplement: Supplementary file 1 — Supplementary Information. [file 41598_2021_4389_MOESM1_ESM.docx]

**Supplementary Information**

**Entropic topography associated with field-induced quantum criticality in a magnetic insulator DyVO_4_**

Dheeraj Ranaut and K. Mukherjee

School of Basic Sciences, Indian Institute of Technology Mandi, Mandi 175005, Himachal Pradesh, India

1. **Crystal Structure**

Rietveld refinement of the XRD pattern obtained at 300 K shows that DyVO_4_ crystallizes in the tetragonal structure with space group *I 41/a m d* and is in single phase (Fig. S1).


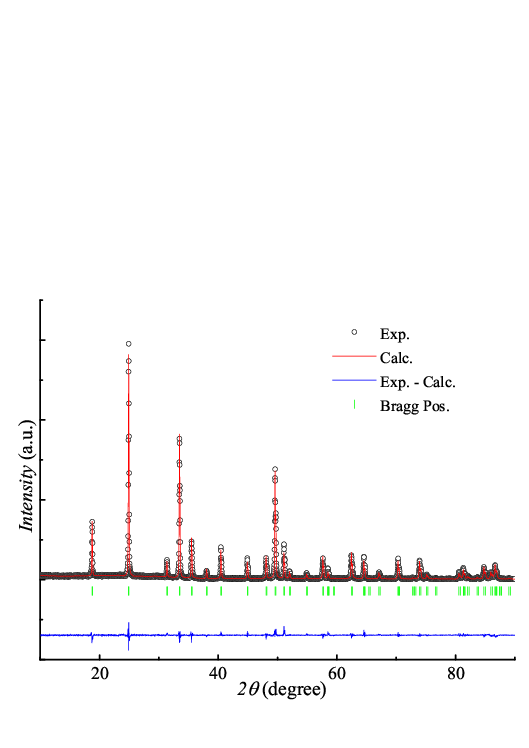


**Fig. S1** Rietveld refined powder x-ray diffraction pattern of DyVO_4_ at 300 K. The open circles indicate the experimental data, while the Ritveld refined pattern is shown by red solid line. The difference curve and Bragg positions are denoted by blue line and green vertical bars, respectively.

1. **Internal magnetic field (*H_i_*) measurements:**

In case of samples with large magnetization, the demagnetization factor needs to be taken in account in order to gain more insight into the field induced magnetic transitions. In polycrystals, generally field induced transitions appear very broad and not much intrinsic information can be gathered. Taking the demagnetization factor results in very sharp transitions, giving more intrinsic information about the system [Ref. 30]. Therefore, in order to get more intrinsic information of the effect of applied magnetic field on magnetic transitions, we have considered demagnetization factor. Thus, the actual magnetic susceptibility (*𝜒_a_*) and internal magnetic field (*H_i_*) has been calculated using the value of demagnetization factor with following relations:

*H_i_* = *H* – *NM* ……. (1)

where, *H_i_* is the internal magnetic field experienced by the sample, *H* is the applied external magnetic field, *N* and *M* are the demagnetization factor and magnetization, respectively.

Hence, we can write:

1/ *𝜒_a_* = 1/ *𝜒* – *N* …….. (2)

where, *𝜒_a_* (=*M*/*H_i_*) is the actual magnetic susceptibility and *𝜒* (=*M*/*H*) is the measured magnetic susceptibility.

Fig. 1(a), 1 (b), 2 of the main manuscript are plotted with respect to *𝜒_a_* and H_i_. Further, it is noticed that within the AFM state, *H*_i_ increase as *T* is lowered; as at lower temperatures the magnetic phase becomes more stable. Fig. S2 illustrates different *M* (*H*_i_) curves at different *T* within the AFM state and it is clearly visible that *H*_i_ increases as *T* decreases (shown blue the blue solid arrow).


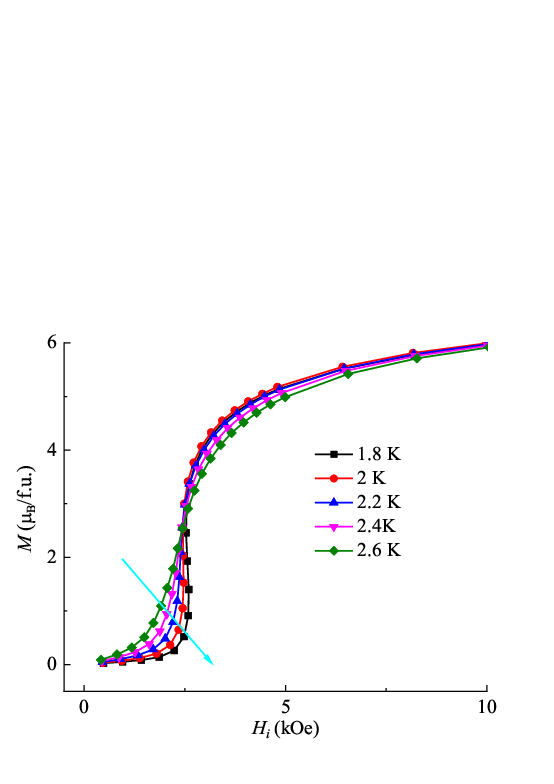


**Fig. S2.** *M* (*H_i_*) curves at different temperatures within the AFM state. The blue arrow shows that as temperature is lowered, the *H_i_* increases.

**3) Magnetocaloric Effect (MCE)**

MCE in temperature range of 1.8 - 5 K and up to a field of 10 kOe, is shown in Fig. S2. An interesting observation is the effect of field on the -Δ*S*, below the *T_N_*. It is noted that below 3 K, the magnitude of the MCE first increases with field up to 5 kOe and then starts decreasing with increasing field. This unusual behavior within the AFM state is believed to be due to the presence of metamagnetic transition with a critical field around 5 kOe. Also, it is observed that the curves become more prominent at higher field around the AFM transition.


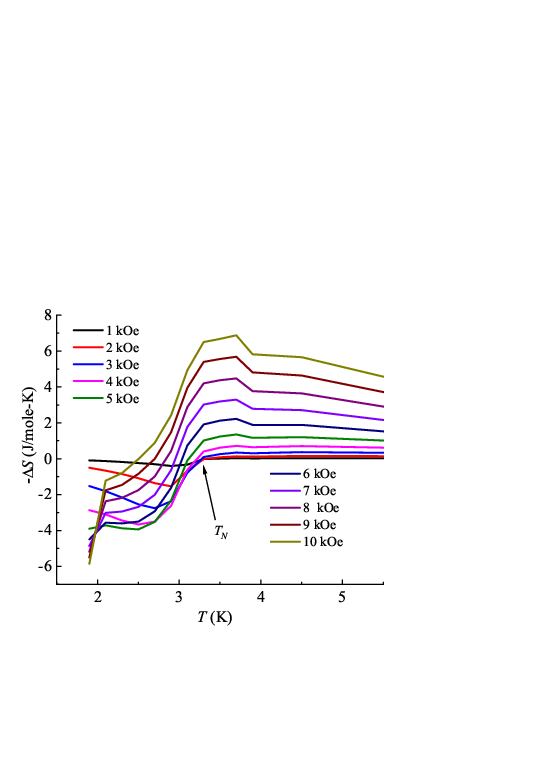


**Fig. S3.** Entropy change, ***-***Δ*S* plotted as function of temperature (below 5 K) for magnetic fields (1–10 kOe). The arrow shows the transition temperature.
